# Supplementary material for: Single molecule poly(A) tail-seq shows LARP4 opposes deadenylation throughout mRNA lifespan with most impact on short tails
Source: eLife. 2020 Aug 3;9:e59186. doi: 10.7554/eLife.59186 (PMC7413741; doi:10.7554/eLife.59186)
Supplement: Supplementary file 6. [file elife-59186-supp6.docx]

**Mattijssen et al.,**

**Supplementary Table ST5.**  DNA oligo probes used for RNA detection, probes were mixed and end-labeled with ^32^P. Hybridization buffer for antisense oligo’s is 6 x SSC, 2 x Denhardt’s, 0.5% SDS and 0.1 mg/ml yeast total RNA. Hybridization was carried out overnight.

| Target | Sequence 5’🡪3’ | Hybridization temp. |
| --- | --- | --- |
| mI8S | CCCACACTGTATTTGGTGTC | 60 **°**C |
| Flag-Hind | AAGCTTGTCGTCATCGTCTTT | 47 **°**C |
| GFP | CGTGCTGCTTCATGTGGTC  GTTCACCAGGGTGTCGCC  GGTCACGAACTCCAGCAGG | 50 **°**C |
| B-globin | CACCAGCCACCACCTTCTG  GGCAGCCTGCACCTGAGG  GCACCTTCTTGCCATGAGCC  CCTCACCACCAACTTCTTCCACATTC  GACAACCAGCAGCCTGCCCAG  CTTAGGATTGCTCATAACAGCATG | 50 **°**C |
| hISG15 | CGCCAGCATCTTCACCGTCA  CTGCTCAGGGACACCTGGA  CTGCGCCTTCAGCTCTGACA  AAGGCGTGCACGCCGATCT  ACCAGCAGGACCGTGCTG  CAGAGGTTCGTCGCATTTGTCC  CGGCCCTTGTTATTCCTCACCA  GTCAGCCGTACCTCGTAGG  CACTTGCTGCTTCAGGTGGG  AGGTCGTCCTGCACACCCT | 51 **°**C |
| hIFIT1 | CCCACACTGTATTTGGTGTC  TTCAGGGCTTCCTCATTCTG  CCTCACATTTGCTTGGTTGTC  CTGCCAGTCTGCCCATGT  GTCCAGGTAAGTCTGGGC  CTTCTTGCAAATGTTCTCCACC  CCATTCTATAGCGGAAGGGA  CACACTTCAGCAAGGCCC  CAGCGCTGGATTCAGGGT  GCCCTATCTGGTGATGCAG | 48 °C |
| hGAPDH | ATGAGGTCCACCACCCTGTTGCTGTAGC  CATCAGCAGAGGGGGCAGAGATGATGAC  CTTCTCCATGGTGGTGAAGACGCCAGTG  GAGTTAAAAGCAGCCCTGGTGACCAGGC | 60 °C |
| mISG15 | CGACTCTCAGAGTCAGAGAC  GCTGCTTGCTCTCACAGTCA  TGCTGCAGGCTCTAGCTCTA  CCCAGCATCTTCACCTTTAGG  CTGGGCAATCTGCTTCTTCAG  CTGTACCACTAGCATCACTGTG  TCAGAAAGACCTCATAGATGTTGCT  TTCGTGGACTTGTTCCCGCT  GGCGCAAATGCTTGATCACTG  CATCTTTTATAACCAACACTGGCTC | 50 °C |
